# Leveraging Large Language Models for Understanding Fundamental Principles of Catalysis

**DOI:** 10.1021/acs.jpcc.6c02607

**Published:** 2026-07-16

**Authors:** Shane S. Michtavy, Sinhara M. H. D. Perera, Marc D. Porosoff

**Affiliations:** Department of Chemical and Sustainability Engineering, 6927 University of Rochester, Rochester, New York 14627, United States

## Abstract

Heterogeneous catalysis presents a distinct challenge for artificial intelligence (AI). Data sets are often small and inconsistently reported, catalyst representations are not standardized, and extracting fundamental knowledge requires integrating performance data, spectroscopic characterizations, and mechanistic models across multiple scales. Language offers a unifying representation across these modalities, making catalysis well suited for leveraging large language models (LLMs). By standardizing how catalytic data is represented, LLMs make dispersed experimental results more accessible to downstream statistical modeling. In this perspective, we focus our discussion around three opportunities where LLMs can significantly contribute to catalysis: (1) text to properties; (2) text to structure; and (3) text to mechanistic models. The discussion is followed by a perspective section on LLM-readiness of data, aligning LLM outputs with scientific correctness, and bridging lab-scale discovery to industrial deployment. Across each area, the most productive applications couple dispersed chemical knowledge with physics-grounded validation to produce verifiable hypotheses and actionable representations.

## Graphical abstract

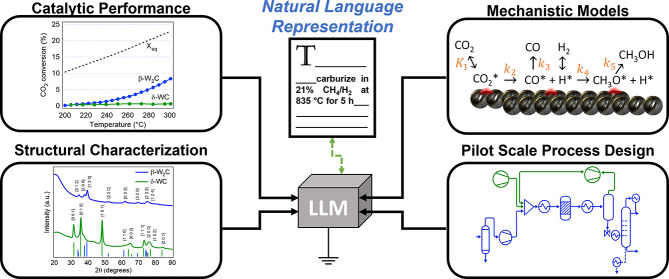

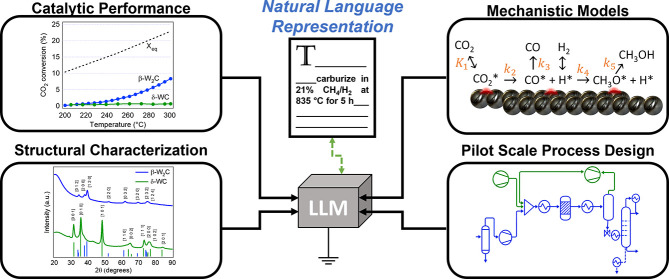

## 1 Introduction

Governments and research institutes worldwide are increasingly treating AI-enabled science as a strategic priority. In Europe, initiatives such as RAISE (Resource for AI Science in Europe) aim to coordinate shared computational infrastructure and AI resources across research institutions.^1^ In Asia, programs in Japan, Singapore, and China are advancing AI-for-science platforms and domain-specialized models.^2–4^ In the United States, the Genesis Mission calls for domain-specific models and AI agents that can generate and test hypotheses, automate research, and compress the time scale of materials development by orders of magnitude. The Genesis Mission frames AI as the cutting-edge research infrastructure that links data, simulations, and automated experimentation to accelerate discovery.^5^

Biomedicine is the clearest and most successful example of AI-driven discovery to date, reflected by the significant amount of private investment in this space.^6,7^ The widespread applications of AI in biomedicine are in part because the well-structured data, prediction targets, and validation frameworks are an ideal fit for statistical learning methods. The success of AlphaFold demonstrates that deep learning can infer three-dimensional protein structures from amino acid sequences with high accuracy, work recognized by the 2024 Nobel Prize in Chemistry.^8^ Researchers have since used AlphaFold to predict more than 200 million structures, roughly 3 orders of magnitude beyond the ca. 200,000 in the Protein Data Bank.

In the physical sciences, the powder crystallography challenge is an example where generative deep learning models simulate accurate crystalline structures directly from powder X-ray diffraction (PXRD) patterns,^9^ and large-scale graph-network screening has expanded the catalog of known stable inorganic crystals by orders of magnitude.^10^ Other examples are flow- and diffusion-based models, which are used for solving and refining inorganic crystal structures from PXRD data with high match rates, cutting per-structure solution time to about 1 s, several orders of magnitude faster than conventional quantum-mechanical or manual pipelines.^11^ Together, these successes in biomedicine and the physical sciences illustrate that generative models are moving beyond narrow predictions and toward widespread use in scientific workflows.

More recently, the emergence of large language models (LLMs) has disrupted AI-for-science workflows because of their applicability across a broad set of input parameters. Domain-specific machine learning (ML) models are typically built around an analytical technique or signal (e.g., protein sequence, diffraction pattern, or molecular geometry), whereas LLMs operate across literature, code, tables, experimental protocols, and structured/unstructured databases. Language offers a unifying representation across these modalities, and leveraging LLMs may enable automated extraction and standardization of heterogeneous data from the literature to facilitate downstream statistical modeling (Figure 1). The value of LLMs therefore lies not only in generation, but also in retrieval, reasoning, planning, and tool use, which together make them an exciting foundation for agentic discovery. Agentic discovery uses agents that unify reasoning, retrieval, generation, and experimental planning, extending AI-driven workflows into domains such as catalysis that have been late adopters of AI.^12–15^

**Figure 1:**
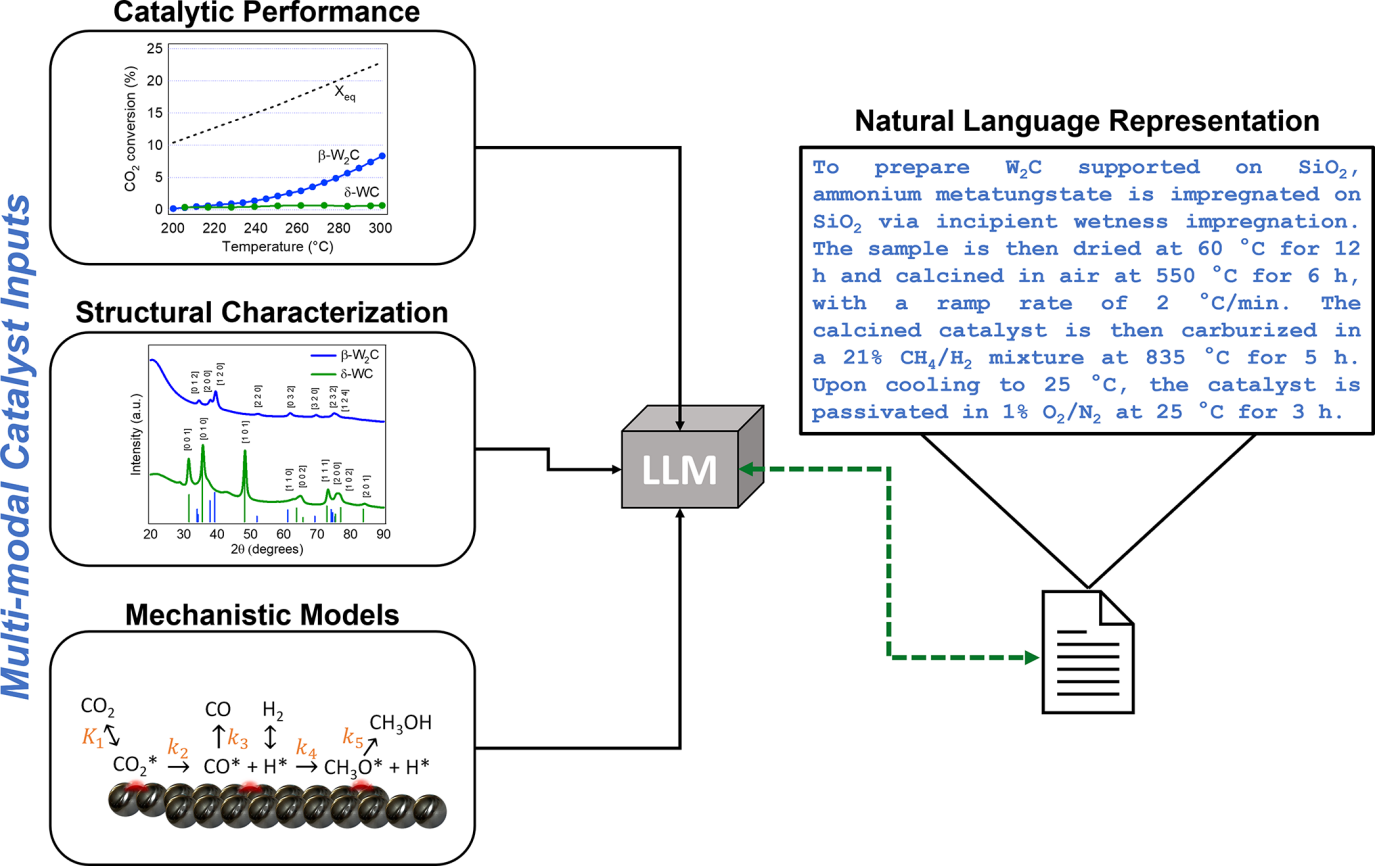
Language as a common representation for heterogeneous catalysis. Catalytic performance, structural characterization, and mechanistic models can each be expressed with language, enabling integration within a shared language-based representation.

Catalysis is a challenging domain for AI-centric workflows because data sets are often small, heterogeneous, and reported with inconsistent parameters and synthesis conditions. Experiments are expensive and slow to validate, and thus model development faces reproducibility issues that may stem from mismatches between experimental, computational, and theoretical descriptions.^16–18^ The same catalytic system can be described through a multitude of inputs including: formulations, synthesis procedures, microscopy images, spectra, adsorption energies, kinetic parameters, reactor conditions, mechanistic hypotheses, simulation outputs, etc. Therefore, a major challenge is that no single representation method serves as a comprehensive ground truth. For example, operando spectroscopic measurements are often method and/or apparatus dependent and lack uniform reporting conventions that might be important for understanding and accurately interpreting data (e.g., thermocouple placement, reactor diameter, beam intensity, etc.).^19^

Language-based workflows are a significant opportunity to rethink how we approach and represent catalysts, potentially making the field more amenable to AI. Natural language is the medium by which disparate results and representations are named, related, interpreted, and made commensurable. Although not often explicitly stated, language is the intrinsic medium by which surface structures are linked to synthesis conditions, spectra to mechanistic claims, operating conditions to performance trends, and computational observables to experimental heuristics. Language can therefore be viewed as a universal representation method that connects catalytic descriptors to experimental data, regardless of the source or technique. By coupling LLMs with external tools and other state-of-the-art AI methods, language-based systems can normalize inconsistent reporting, identify when distinct formulations refer to the same material or mechanism, map between symbolic and learned representations, and route scientific questions to the appropriate retrieval, simulation, or validation technique.^20^ The less-realized value of LLMs is that they use language as an interface to make fragmented scientific knowledge accessible and computationally tractable.

A central goal in heterogeneous catalysis is identifying the fundamental principles that link molecular-scale chemical interactions with observable and tunable macroscopic parameters.^21^ Despite decades of work, catalyst development still relies on empirical trial-and-error screening, and most deployed AI models accelerate search rather than deliver mechanistic understanding.^22^ Language models offer a brand new entry point into extracting fundamental knowledge in catalysis because the inputs are concepts, relationships, and mechanistic statements expressed in natural language, rather than numerical representations of atomic structure or electronic potential. Because catalytic parameters such as phase, coordination number, synthesis conditions, reactor type, and temperature are already described in paragraphs of text across the literature, they can be extracted and organized into structured and verifiable hypotheses.^16,17,23^ Unlike methods that operate on preselected numerical features, LLMs impose no constraints on what counts as relevant input. This means incidental but consequential information, such as chemical lot numbers that encode batch-to-batch variability can be captured alongside typically reported experimental parameters.^24^ The result is that experimental nuance becomes actionable and provides motivation for including more comprehensive information within the methods sections of publications.

In this perspective, we evaluate how LLMs can be used to extract fundamental knowledge and add value to the field of catalysis. We divide the paper into three areas: (1) text to properties, (2) text to structure, and (3) text to mechanistic models, before discussing our point of view on the future of LLMs in catalysis. The perspective section is organized around LLM-readiness of catalysis data, verifying LLM predictions with scientific principles including kinetics, thermodynamics, and experimental data, as well as bridging the gap between lab-scale discovery and industrial deployment by encoding scale-dependent synthesis, reactor, and feed information in an actionable, language-based format.

## 2 LLMs for Fundamental Catalytic Knowledge Discovery

Recent work in heterogeneous catalysis demonstrates successes in data processing with LLMs through literature mining, schema-guided extraction, and knowledge-graph construction, which convert published text, tables, figures, and spectra into machine-readable databases for downstream modeling.^16,23,25^ As LLMs have become increasingly more capable, they are paired with closed-loop autonomous experimentation and multiscale workflows that connect theory, experiment, and uncertainty-aware reasoning for catalyst design.^16,26–28^ Because of the potential for LLMs to significantly impact the field, we have organized the use cases of extracting fundamental catalytic knowledge around three areas likely to have a lasting impact:

1. **Text to properties:** Interpreting structure–property relationships is a cornerstone of catalysis. This task is naturally framed as an inverse design problem, an area where LLMs are well-suited to excel because of the facile interpretability of the natural language outputs.
2. **Text to structure:** Forward design of catalysts is a natural extension of LLMs, where chemical descriptions, synthesis procedures, and spectra can be input into an LLM and translated into physically valid candidate structures for enabling improved hypothesis-driven design.
3. **Text to mechanistic models:** Organizing heterogeneous experimental data into coherent reaction networks is an emerging application for LLMs, which show potential for translating unstructured literature data into hypotheses about the reaction mechanism and kinetic parameters.

### 2.1 Text to Properties

Elucidating structure–property relationships in catalytic systems can be framed as an inverse design problem. Catalyst features (e.g., activity, selectivity, and stability) are specified as desired outcomes, and the task of researchers in the field is to identify catalyst structures and reaction conditions that achieve these outcomes.^29^ A major bottleneck for property prediction with LLMs is data sparsity, because fine-tuning typically requires hundreds to thousands of labeled examples, yet a reaction experiment rarely yields more than a few data points that characterize reaction rate, selectivity, and stability.

Bayesian optimization (BO) and related closed-loop AI workflows are a potential solution because they are explicitly designed for low-data, high-cost experimental regimes.^30^ By iteratively selecting experiments that maximize expected improvement with respect to a probabilistic surrogate model, BO can identify high-performing candidates with fewer experiments than random screening.^30,31^ In a self-driving catalysis study, BO identified near–optimal reaction conditions in an active-learning campaign on the FeCoCuZr catalyst family. In this example, 86 targeted experiments identified catalyst compositions and reaction conditions that increased alcohol productivity while minimizing undesired CO_2_ and CH_4_ formation, achieving more than a 90% reduction in cost and environmental impact relative to conventional trial-and-error screening.^31^

Recent work has combined BO with in-context learning (BO-ICL), in which pretrained LLMs are surrogate models that are used off-the-shelf without fine-tuning.^32–34^ Rather than training a task-specific model, BO-ICL uses a small set of experiments and the associated outcomes (labels) as context within a prompt, and leverages the extensive and diverse pretraining of LLMs to guide predictions (Figure 2). Candidate systems are described through synthesis and testing procedures expressed in natural language, reducing the need for explicit feature engineering.^32^ This property-first approach prioritizes optimization of target properties before detailed characterization, effectively inverting the conventional experimental workflow and potentially saving significant resources characterizing suboptimal materials.^32^ On standard benchmarks, including aqueous solubility and oxidative coupling of methane, BO-ICL matches or outperforms Gaussian process (GP) surrogates.^32^ In live experiments on the reverse water–gas shift reaction, the BO-ICL method identifies multimetallic catalysts approaching equilibrium CO yield within six iterative cycles from a candidate pool of 3700 catalysts, and within ten iterations from a pool of 360,000 trimetallic catalysts composed of formulations that may be nonobvious to an expert in the field.^32^

**Figure 2:**
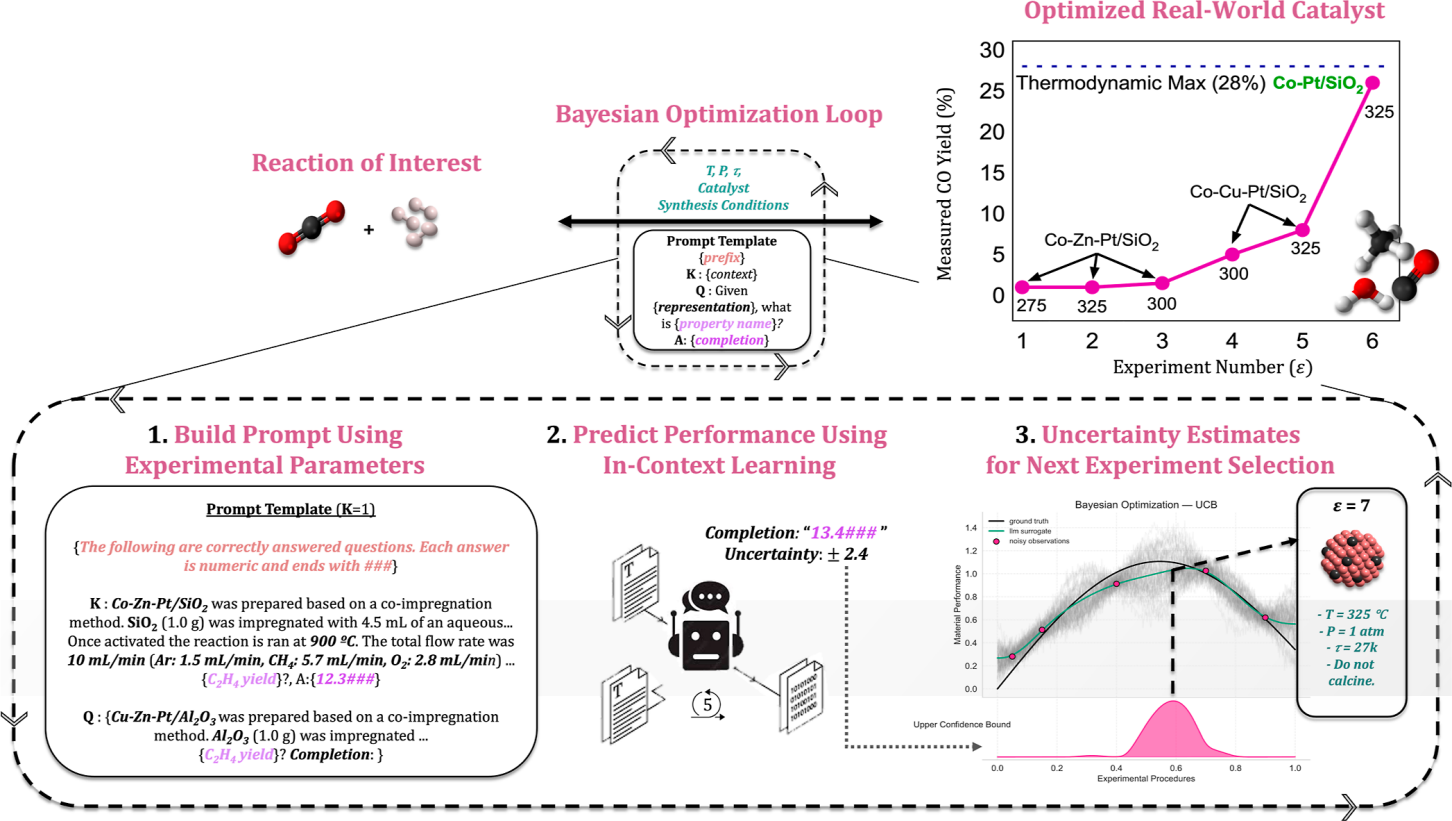
A high-level overview of the closed-loop Bayesian optimization with in-context learning (BO-ICL) that uses natural language to represent a catalyst design space for efficient exploration. The workflow involves conversion of tabular data into an experimental procedure, which incorporates both synthesis and reaction parameters. BO-ICL leverages well-established BO techniques to efficiently identify actionable experimental conditions that maximize a desired objective function, while leveraging in-context learning to eliminate the need for extensive model pretraining. In this figure, we highlight a success case of BO-ICL by our group for optimizing trimetallic catalysts for selective CO_2_ conversion to CO. Reproduced from ref 32. Copyright 2026 American Chemical Society.

A practical advantage of BO-ICL over conventional Gaussian process-based BO is that qualitative experimental context, including synthesis procedures, pretreatment conditions, and support identity can be expressed directly in natural language rather than manually encoded into numerical descriptors.^35^ By leveraging LLMs as a tool for processing language-based representations of catalysts, researchers can directly use their own data, protocols, and procedures without translation into a structured feature space. The oxidative coupling of methane (OCM) and reverse water–gas shift (RWGS) examples with BO-ICL illustrate this point because the catalyst representations are solely synthesis procedures and reaction conditions without any engineered descriptors. A Gaussian process based on composition alone would not capture the same information without substantial hyperparameter tuning.^36^

The next frontier is interpretability of the results, e.g., understanding why an LLM selects a particular experiment. LLM-guided approaches can efficiently identify high-performing candidates, but the reasoning behind these predictions is often difficult to verify.^37^ Because LLMs are effectively black-box models, the physical relationships encoded in their internal representations are not generally transparent, and this limitation persists even when the models are used to rank candidates or provide a line of reasoning.^38,39^ Even with the recent rise of reasoning models, which are prompted to justify their decisions, the accuracy of the chain-of-thought is largely unverifiable. Approaches that combine LLMs with interpretable machine learning offer opportunities to understand and test why a particular binding energy, coordination environment, or oxidation state affects catalytic performance, thereby shifting LLMs from search tools toward hypothesis generators.^40,41^ In this sense, LLM-generated hypotheses represent a new form of physical descriptors that can be applied, tested, and refined on catalyst systems.

### 2.2 Text to Structure

A central challenge in catalyst design is not generating structures, but generating the right structures. Activity, selectivity, and stability depend on both atomistically precise and coarse features such as phase, coordination number, active environment, defect chemistry, cation ordering, and support–active-site geometry that are often expressed in natural language.^42–46^ For example, M1-phase Mo–V oxides, oxygen-deficient perovskites, and FCC-coordinated adsorbates carry precise structural meaning, but conventional atomistic modeling pipelines cannot accept these descriptions as direct inputs. In practice, the text supplied to generative models spans a wide range of inputs from a simple composition (Pt_3_Ni) to more complex formulas that may include space groups and lattice parameters (orthorhombic M1-phase Mo–V–Te–Nb oxide). These varied inputs also expose a characteristic problem because compositions are often underspecified. For example, W_*x*_C maps to several polymorphs (e.g., δ-WC, β-W_2_C, and γ-WC_1–*x*_) with distinct structures and catalytic behavior, so a composition-only prompt maps to multiple valid structures without an encoded phase or space group.^47^ Text-to-structure workflows are beginning to close the gap between molecular models and natural language-based descriptions, and by coupling language with physics-aware geometry, text-based descriptions can function as compact and transferable representations of chemical expertise.^48–50^

Recent text-to-structure methods are moving toward separation of two entangled tasks: interpreting chemical meaning from language, and producing physically valid three-dimensional structures.^51,52^ LLMs trained on literature and multimodal data can encode transferable chemical concepts such as phase, coordination, and heats of adsorption. From a natural language prompt, these models can narrow the search to a specific region of composition and symmetry space.^50,53^ Fine-tuned LLMs can even output inorganic crystal structures directly as text, producing stable candidates without an explicit geometry decoder, but at the cost of weaker physical constraints.^54^ Complementing these workflows, MatLLMSearch uses pretrained LLMs embedded in an evolutionary search loop to propose novel, stable crystal structures without any fine-tuning, indicating that chemically useful generative priors are already latently embedded within general-purpose LLMs.^55^ What LLMs cannot do reliably is convert these constraints into physically valid coordinates and lattice parameters. Translation requires models that treat rotations, translations, and permutations as hard physical constraints rather than learned approximations.^51,53^ The most capable text-to-structure systems are hybrid models that use an LLM to narrow the search to chemically meaningful regions of structure space, and then use an equivariant or flow-based geometry engine to produce three-dimensional coordinates that satisfy those constraints.^50,51,53^ Recent systems including Mat2Seq,^56^ Lang2Str,^57^ EquiLLM,^48^ GeomCLIP,^58^ Chemeleon,^49^ and CrysText^50^ reflect this division of labor to varying degrees, differing primarily in where the language–geometry interface is drawn and how tightly the two modules are coupled.

Before these hybrid systems can generalize reliably, they must resolve a representational problem. Standard Crystallographic Information File (CIF) descriptions of the same crystal are not unique, producing many different token sequences for an identical structure. Mat2Seq addresses this gap by generating canonical, SE(3) and periodically invariant sequences, as shown in Figure 3, ensuring that equivalent crystal descriptions map to the same representation without augmentation.^56^ Here the “text” is a serialized crystal form that can be used to reconstruct the full structure from the formula, space-group, lattice parameters (*a*, *b*, *c*, α, β, γ), and symmetry-irreducible atoms with their fractional coordinates.^56^ Canonical representations of accurate crystal structures like those in Mat2Seq are a prerequisite for language models to learn consistent structure–property relationships across the composition space.

**Figure 3:**
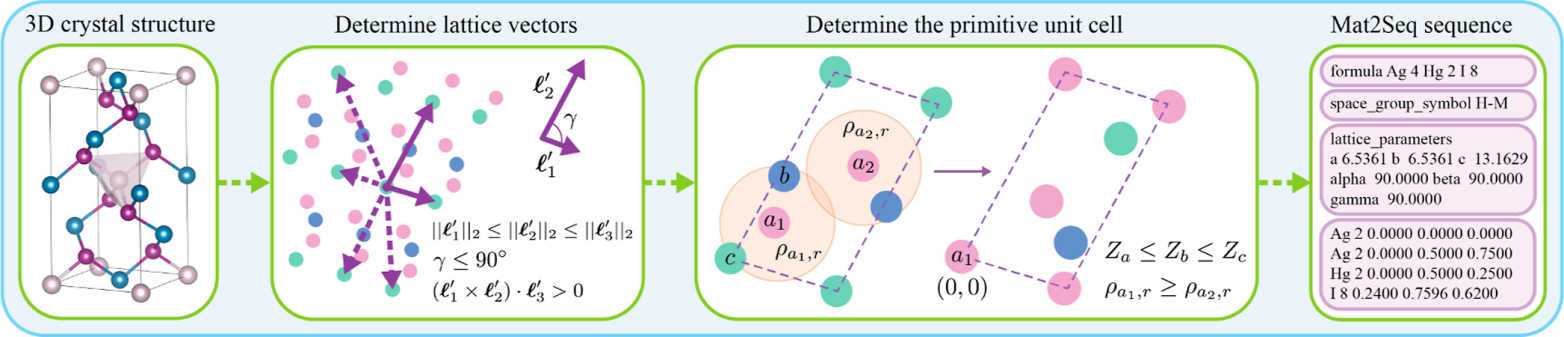
Language-guided crystal structure generation via Mat2Seq. Mat2Seq converts 3D crystal structures into unique, SE(3) and periodically invariant 1D sequences, enabling language models to generate and reason over crystal structures without ambiguity from nonunique CIF representations. The pipeline proceeds from structure to canonical lattice vectors, primitive unit cell, and invariant sequence. Reproduced from ref 56 under the terms of a Creative Commons Attribution–NonCommercial–ShareAlike 4.0 (CC BY-NC-SA 4.0) license. Copyright 2025 The Authors.

Lang2Str then can take a short textual description of the unit cell structure (e.g., GaTe space group 194 consisting of eight atoms), and expand it into a more detailed description that captures coordination numbers, nearest neighbors, and bond lengths in a format amenable to a conditioned normalizing-flow model that maps the description into lattice parameters and atomic coordinates.^57^ The benefit of separating language from geometry is demonstrated via the MP-20 benchmark, where Lang2Str matches or exceeds flow- and diffusion-based baselines, reaching a stable, unique, and novel (S.U.N.) rate of 3.2%, that increases to ca. 6% with rejection sampling. Furthermore, ablation studies show that the natural language description provides richer conditioning than space-group labels alone, demonstrating the utility of using language-based descriptions for catalyst representation.^57^ Lang2Str is conceptually significant for catalysis because it can also incorporate reaction-level descriptors such as preferred N_2_ activation pathway, relative stability of OOH* versus O*, or proximity to the maximum of a volcano plot, leading to proposed structures optimized for a target reaction rather than a target geometry.^57,59,60^ Direct experimental validation of reaction-conditioned structure generation has not yet been demonstrated, but the architecture of Lang2Str is a blueprint for language-guided, physically constrained exploration of catalyst structures.

Although Lang2Str can propose candidate structures, it does not address computational validation. Once a candidate structure is proposed, the pathway from geometry to energetics still requires convergence testing, error handling, and uncertainty estimation that generative models do not attempt. DREAMS (DFT-based Research Engine for Agentic Materials Screening) addresses this gap by leveraging a hierarchy of LLM agents to plan and execute full density functional theory (DFT) workflows with minimal human intervention, converging plane-wave cutoffs and *k*-point sampling, generating adsorbate configurations, recovering from Self-Consistent Field (SCF) convergence failures, and quantifying uncertainty.^60^ On the Sol27 lattice-constant benchmark, DREAMS achieves average errors below 1% relative to experimental reference values.^60^ More relevant to catalysis, DREAMS reproduces expert-level DFT ordering of CO adsorption energies across sites and orientations on Pt(111), favoring the FCC site by roughly 0.12 eV at the GGA level, with a Bayesian error estimation functional (BEEF-vdW) ensemble standard deviation of about 0.01 eV.^60^ DREAMS is therefore a noteworthy tool because it reveals that computational catalysis methods encoded in scientific text can be extracted and operationalized by LLMs.^60^

Although LLMs have been used in recent and exciting applications for structure generation, they are not positioned to replace physics engines in the near term. Emerging approaches such as reinforcement learning with verifiable rewards (RLVR) may eventually tighten language–physics integration, but equivariant models, machine-learned potentials, and DFT remain essential for structural refinement, energetic validation, and uncertainty estimation.^51,61^ The more plausible role of LLMs is converting text into structured, verifiable hypotheses that can be evaluated with physics-based pipelines.^60^ Phase labels, coordination environments, defect patterns, and reactivity descriptors are the language of catalytic reasoning, but they are not native inputs to most atomistic workflows.^43,62^ The most productive role for LLMs is a semantic interface that organizes the search through structure space using chemically interpretable priors, while physics-aware models provide the generative and evaluative backbone. Language then becomes a practical medium for encoding and deploying catalytic expertise at scale.^53,56^

### 2.3 Text to Mechanistic Models

Mechanistic models link molecular-level insight into macroscopic behavior by translating a hypothesis into a network of elementary steps, each parametrized by thermodynamic and kinetic quantities derived from potential energy surfaces, experimental measurements, or both.^63^ Because the parameters in these models correspond to physical quantities rather than fitted coefficients, they can be transferred from one system to another and used alongside reactor models to predict observable performance.

A typical workflow for elucidating a reaction mechanism proceeds in three stages: (i) proposing candidate reaction networks from chemical intuition, literature precedent, known surface motifs, and more recently, automated extraction from published papers;^16,64^ (ii) screening and refining pathways using thermodynamic and kinetic constraints, supported by DFT and transition state theory; and (iii) solving the resulting coupled rate equations to predict observables such as turnover frequency, selectivity, coverages, and apparent activation barriers.

In practice, the bottleneck in developing a mechanistic model is not solving the rate equations, but constructing the reaction network, identifying plausible intermediates, and generating thermodynamically consistent parameters. Even for relatively simple systems, these steps require repeated iteration between spectroscopy, kinetics, and computation.^63,65^ As system complexity increases, the number of plausible pathways expands exponentially, and the usefulness of a model depends on whether the initial mechanistic search was sufficiently broad and whether the final model is tightly constrained by observable physical parameters.

Language models are well-suited for the early stages of mechanistic model development, where the goal is organizing dispersed literature into testable reaction networks.^25,37,64,66,67^ In heterogeneous catalysis, experimental data and results become interpretable only when linked across the entire catalytic process from synthesis to characterization to reactor studies. Synthesis protocols determine the active site structure, thereby constraining mechanistic pathways, which in turn manifest as observable performance.^64^ These relationships are difficult to recover when the literature is parsed as data points without the appropriate experimental context.

AgentCAT addresses this problem by combining schema-governed extraction with a dependency-aware reaction-network knowledge graph to link catalyst identity, active site descriptors, mechanistic claims, supporting evidence, and macroscopic outcomes. AgentCAT performs the upstream extraction and structuring task rather than generating complete candidate mechanisms, and the result is a corpus of literature-grounded inputs that can seed downstream reaction network construction more efficiently and comprehensively than an individual researcher’s intuition.^64^ The workflow has been applied across approximately 800 peer-reviewed chemical engineering publications in catalysis, converting fragmented literature into structured inputs that seed reaction network construction, as shown in Figure 4.^64^ The candidate reaction mechanisms that emerge from this construction still require validation against thermodynamic constraints, kinetic measurements, and experimental observables, but they provide a starting point that is broader and more systematic than manual literature review.^37,66^

**Figure 4:**
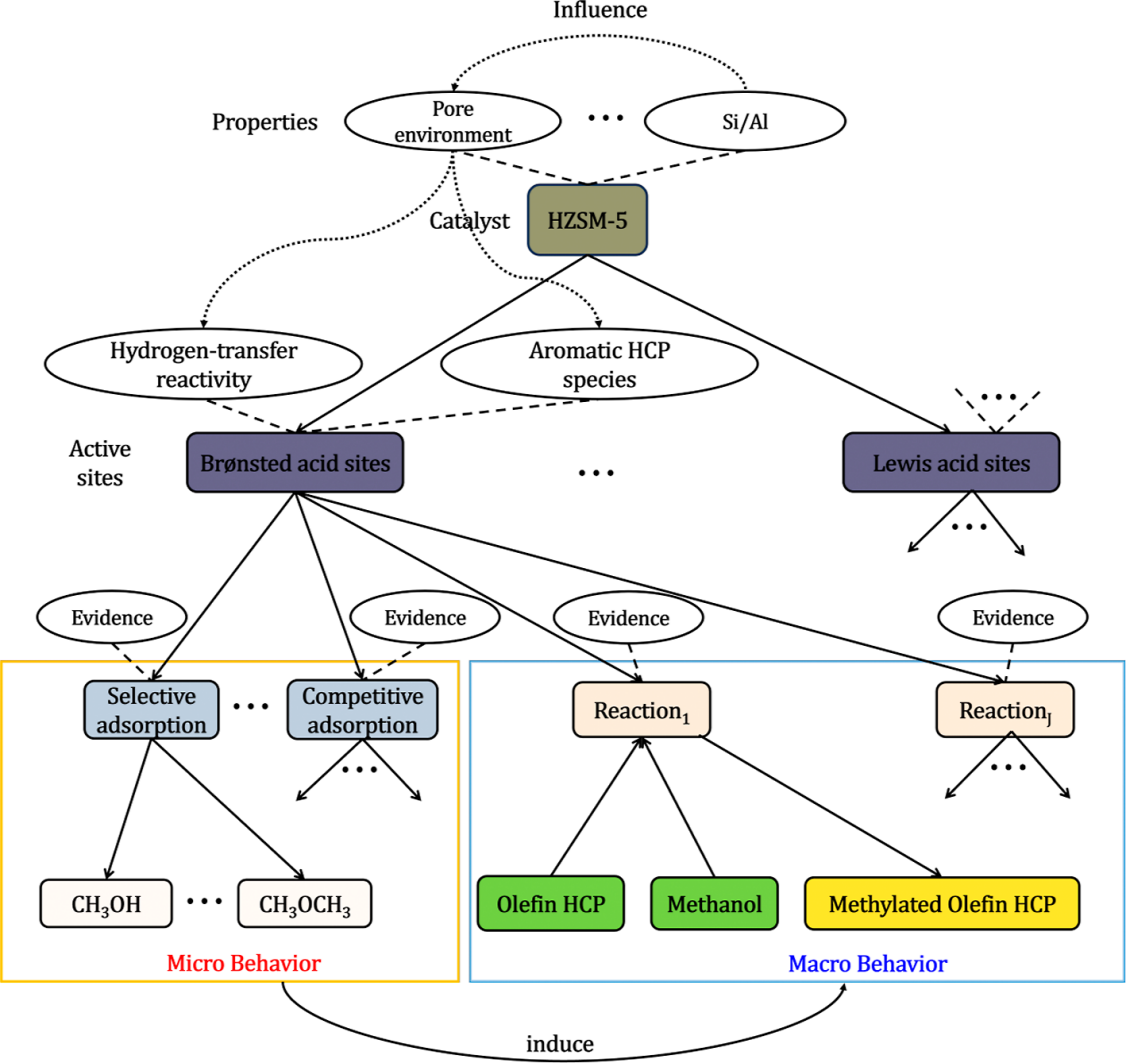
How mechanistic events on catalyst active sites and within pore environments propagate to macroscopic reaction outcomes in the H-ZSM-5 catalyzed methanol-to-olefins reaction. Synthesis-controlled descriptors such as Si/Al ratio determine active-site properties, which constrain mechanistic pathways and ultimately manifest as observable selectivity and conversion. This synthesis–structure–performance relationship motivates language-based extraction approaches that link experimental details of the catalytic process rather than treating them as isolated data points. Reproduced from ref 64 under the terms of a Creative Commons Attribution 4.0 (CC BY 4.0) license. Copyright 2026 The Authors.

Convincing end-to-end demonstrations of automated reaction network construction are limited. There are few studies showing an LLM directly generating, validating, and refining a complete microkinetic model for a heterogeneous catalytic reaction.^37,68^ Current approaches are modular and poorly integrated, with one component literature mining, another handling quantum-chemical screening, and another fitting parameters.^25,37,66^ ChemReasoner is a more capable model that couples language-guided search with quantum-chemical feedback, showing that mechanistic hypotheses become more useful when evaluated against energetic and structural signals rather than text alone.^40^ More broadly, domain-adapted agentic frameworks are beginning to automate portions of the multiscale modeling pipeline, from mechanism generation and DFT execution to model refinement and sensitivity analysis, while using language as both an information interface and a reasoning scaffold.^37,40^

Overall, language may change the entry point and lower the barrier to mechanistic modeling. Rather than requiring a fully formed reaction network from the outset, researchers can begin from dispersed prior knowledge and systematically reorganize that knowledge into testable hypotheses. Because mechanistic models are expressed in terms of elementary steps and transferable parameters, insight derived in one context can be carried across catalyst families, reaction conditions, and modeling frameworks. Language does not replace physics, but it makes the path toward physics-informed modeling more systematic and accessible.

## 3 Perspective

LLMs excel at pattern recognition, but they are not grounded in thermodynamics or kinetics, the physical laws that govern catalysis. LLMs have not yet seen widespread adaptation in catalysis because physical correctness is not enforced. Domain expertise carries no more weight than language, and the training process rewards fluency rather than accuracy (e.g., incorrect answers can sound correct). The solution is not to wait for larger and more capable models, but to embed existing LLMs within frameworks that enforce physical constraints. To clearly outline the future challenges and opportunities of LLMs in catalysis, we organize the perspective section around three key ideas: (1) representing catalysis data in an LLM-ready format to ensure comparisons reflect physical phenomena; (2) verifying model outputs internally by rewarding scientific correctness against physical criteria, and externally by checking outputs against thermodynamics, kinetics, and/or experiments; and (3) extending LLM applications across scales to accelerate catalyst deployment for industrial applications. When all three elements work together, the LLM proposes candidate mechanisms, physically grounded tools verify the hypothesis, and the outcomes are translated across length scales to accelerate deployment and scale-up.

### 3.1 LLM-Readiness of Catalysis Data

Standard LLM training objectives reward fluent, plausible sounding and contextually appropriate responses, but do not require these responses to obey physical constraints. A model that sounds like an expert and a model that reasons like one are trained against different reward functions, and the distinction is critically important in catalysis where correctness is defined by thermodynamics, kinetics, and experimental outcomes.^69–71^

A persistent limitation of LLM-based workflows is the content of the training data, where inconsistent reporting of reaction conditions, catalyst descriptors, and the absence of negative results degrades LLM predictions.^18,72,73^ Models trained on biased corpora tend to reproduce reporting patterns rather than the underlying structure–property relationships that govern catalytic behavior, a failure mode that is difficult to detect from output fluency alone.^15,16^

Data heterogeneity presents a separate methodological challenge that makes uncertainty difficult to quantify and properly address.^16,23,74,75^ A clear example comes from the dry methane reforming literature, where catalyst performance depends on metals, supports, promoters, and synthesis methods, yet inconsistent reporting limits reproducibility and comparability. Across 149 Ni/Al_2_O_3_ studies, only 82 (55%) were found suitable for reuse in data-driven analysis, whereas the remainder were excluded because of missing parameters, inconsistent data presentation, or nonquantitative data presentations from analytical techniques (Figure 5).^76^ Key details such as calcination conditions, reduction protocols, flow rates, and catalyst mass are often incompletely reported, and common characterization methods, including X-ray diffraction (XRD), transmission electron microscopy (TEM), and H_2_-temperature-programmed reduction (TPR), are frequently presented without standardized quantitative outputs. As a result, performance differences across studies may partly reflect differences in reporting, analysis, or experimental protocol rather than intrinsic material variations.^23,74,76^ The absence of a compact, widely adopted catalyst representation analogous to simplified molecular input line entry system (SMILES) for molecular systems further suggests that these challenges will persist without deliberate, community-wide efforts on data and reporting standardization.^43,44^

**Figure 5:**
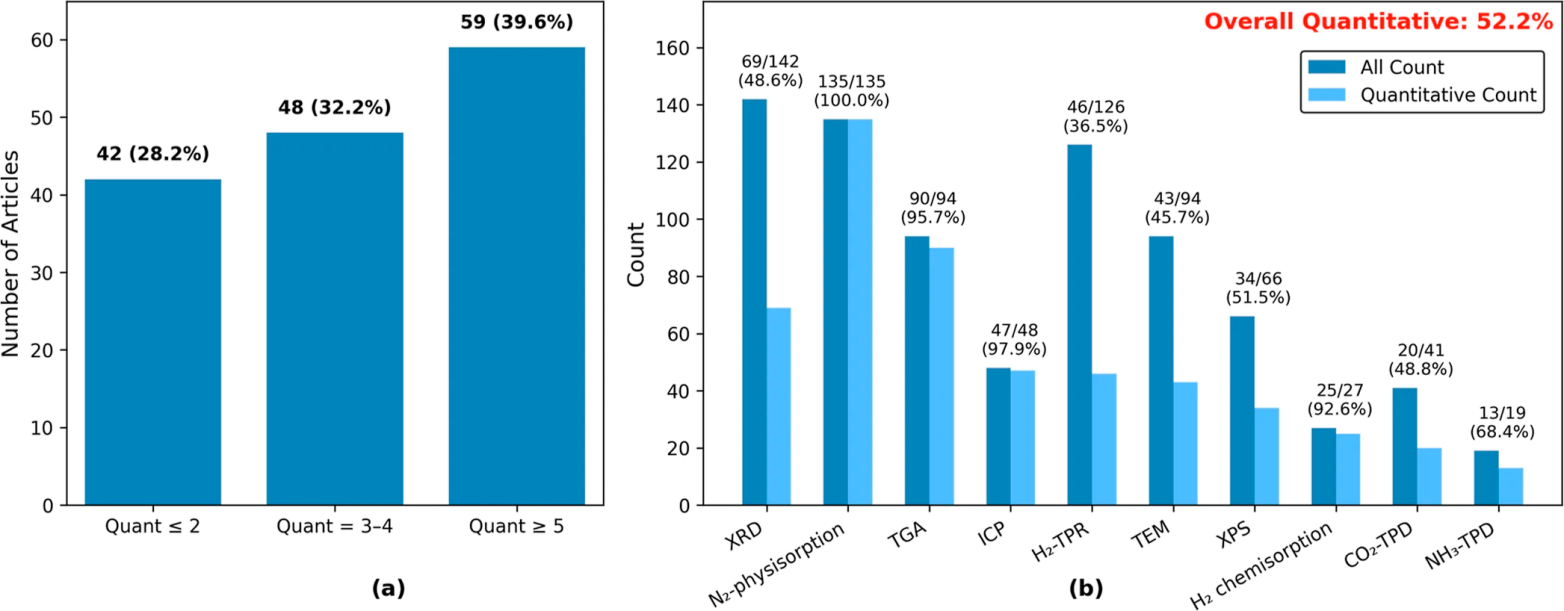
Distribution and comparison of characterization techniques in reviewed dry methane reforming (DMR) studies. (a) Distribution of articles based on the number of quantitative characterization techniques used. The blue vertical bars represent the number of studies falling into three categories: two or fewer quantitative techniques, three to four quantitative techniques, and five or more quantitative techniques. The numbers and percentages above the bars indicate the number of articles belonging to each group, showing that 39.6% of studies report five or more quantitative techniques, while 28.2% report two or fewer. (b) Frequency of the top ten characterization techniques and their respective proportion of quantitative results. Dark blue bars indicate the total number of studies using each technique, while light blue bars indicate the number of studies that reported quantitative values. The percentage values above each pair represent the ratio of quantitative to total counts. The red text “Overall Quantitative: 52.2%” in the upper right denotes the overall fraction of quantitative characterizations among all techniques surveyed. Reproduced with permission from ref 76 under the terms of a Creative Commons Attribution-NonCommercial-NoDerivatives 4.0 International License (CC BY-NC-ND 4.0). Copyright 2026 Springer Nature.

Benchmarking presents another obstacle because many emerging areas of catalysis lack standardized data sets, reliable ground truths, or broadly accepted evaluation metrics that better reflect genuine scientific reasoning, rather than surface-level fluency.^60,75,77,78^ Even general chemistry benchmarks such as ChemBench, which score models against expert chemists, show that strong aggregate scores can mask systematic failures on reasoning-intensive tasks.^79^ As one recent Science news article states,^80^ “what gets measured tends to shape what improves, and in science, optimizing the wrong target can produce outcomes that perform well on well-characterized benchmarks while remaining unreliable in practice”. For LLMs in heterogeneous catalysis, evaluation should therefore be extended beyond benchmark accuracy to include citation fidelity, uncertainty communication, consistency under reformulation, and robustness to incomplete or conflicting evidence.^75,77^

Retraining models on catalysis-specific data is an intuitive solution, but two important barriers limit this approach. First, supervised fine-tuning requires hundreds to thousands of consistently measured examples for a given reaction, and that volume of curated data rarely exists. Instead, lower-complexity statistical models such as decision-tree ensembles or Gaussian processes are often used.^81^ In contrast, language-based representations incorporate both categorical and continuous synthesis variables without feature engineering of descriptors. Second, as discussed above, nonstandardized reporting means the same physical quantity appears in many incompatible written forms, and embeddings trained on general text encode contextual similarity rather than physical meaning, so two descriptions that are physically very different can sit close together in embedding space when they differ by only a token or two.^82,83^ For example, reaction temperatures reported as 620 °C and 620 K differ by 273 K, yet are nearly indistinguishable as embedded strings, which can conflate their similarity in the embeddings that the LLMs use for reasoning.^84,85^ The same problem arises for oxidation states (Fe^2+^ versus Fe^3+^), units (wt % and mol %), and other examples that are routine features of catalytic reporting. A representation that conflates units and other important distinguishing features will propagate physically incorrect matches into retrieval, database comparison, and rewards. Representational alignment should therefore make physically meaningful parameters take precedence over semantics.

The most verifiable route toward ensuring LLMs correctly interpret chemical meaning from similar blocks of text is deterministic extraction and normalization. Here, the LLM first parses physically meaningful quantities, including temperatures, pressures, oxidation states, stoichiometries, and compositions, and converts to canonical units so that 620 °C and 620 K are compared as 893 and 620 K. A complementary approach reshapes the representation through contrastive alignment with automatically generated hard negatives.^86,87^ In this case, physically distant but semantically similar pairs are synthesized by swapping units or oxidation states, and the model is trained to separate them using physics to define positives and negatives without manual labels. A hybrid model in which deterministic normalization supplies both the comparison features and the hard negatives for contrastive training is likely the most robust, keeping the interpretation grounded in verifiable physical quantities rather than learned similarity.

### 3.2 Verification of LLM Predictions for Alignment with Scientific Principles

Hallucinations and context drift compound the data problem when an LLM must track experimental information across complex workflows with many steps, assumptions, and intermediates. Errors such as misidentified intermediates, incorrect stoichiometry, or conflated reaction conditions can propagate and corrupt downstream outputs leading to inaccurate statements and interpretations.^18,73^ Unlike conversational use of LLMs where hallucinations are often immediately obvious, errors in multistep scientific workflows may appear physically plausible, albeit physically inaccurate, making detection reliant on independent validation steps.^16,72^

Two complementary forms of verification can address hallucinations and context drift. Internal verification rewards the model against physically measurable criteria, so invalid reasoning is penalized during model training. External verification references model outputs against domain specific tools, simulations, and experiments. We detail both types of verification below to demonstrate how they improve LLM-based predictions for catalysis.

Reinforcement learning with verifiable rewards (RLVR) is a mechanism of training the model where a proposed intermediate is scored against a computed energy, the candidate pathway is checked for stoichiometry and other physical constraints, and model outputs that fail these checks receive no reward.^69,88^ A practical advantage of RLVR is that it does not require the field to first standardize reporting practices. RLVR can improve model behavior with existing data by encoding catalytic heuristics and physical constraints directly into the reward function.^69,81^ In this framework, the LLM proposes candidate mechanisms, articulates the mechanistic basis for each proposal, identifies the relevant degrees of freedom, and determines which calculations or experiments should be next.^16,72,89^ Whether a proposed mechanism is accepted or rejected is determined externally by physics and experiments, not by an explanation that sounds convincing.

RLVR has been shown to elicit extended, structured reasoning for mathematical and programming tasks.^90^ Whether the same dynamic transfers to mechanistic reasoning in catalysis is plausible but not yet established, and it remains debated whether such rewards instill genuinely new reasoning ability or primarily access latent reasoning already present in the base model.^91^ To the extent RLVR transfers to mechanistic reasoning, repeatedly requiring mechanistic explanations to survive physical audit would bias the model toward physically grounded reasoning patterns rather than fluent ones, shifting its behavior toward that of a domain specialist. Domain-specific models trained in this manner are often better suited to specialized tasks than general-purpose foundation models, though the advantage depends on whether the reward function scores the mechanistic reasoning chain directly or only the terminal output.^81^

A concrete example of internal verification is high-throughput deep reinforcement learning with first-principles (HDRL-FP), which acts as the verifier to supply rewards. HDRL-FP treats atomic migration as a Markov decision process in which states are atomic configurations and rewards are derived directly from potential energy landscapes and first-principles.^92^ The workflow captures hydrogen and nitrogen migration, as well as bond formation during ammonia synthesis on Fe(111), demonstrating relevance beyond simple migration on surfaces.^92^ If used in conjunction with RLVR, a language model proposes a mechanistic hypothesis, which is then evaluated against energy barriers, stoichiometric consistency, and candidate transition states, with thermochemical corrections applied where necessary. By running thousands of concurrent simulations on a single graphics processing unit (GPU), HDRL-FP can converge to reaction pathways with lower barriers than those identified by nudged elastic band calculations, while reducing convergence times from days to under an hour.^92^ This gain in throughput could lower the per-trajectory cost of verification, which makes a loop of proposal, verification, and alignment conceivable at scale.

Although HDRL-FP is a promising method for verifying LLM outputs via DFT calculations, the utility of such a loop depends on its simulated environment, which can be incomplete. The reward signal is constructed from DFT electronic energies, so at the high temperatures relevant to catalysis, it does not yet capture anharmonic contributions to the free energy barriers. The set of reactions tested so far is also narrow; however, we expect the methodology will be generalizable. The representation of reactions on surfaces is defined solely by atomic positions without reaction-specific encoding, an approach already validated across both single- and two-atom migration scenarios with consistent hyperparameters. Each new system also requires the labor-intensive step of building a potential energy landscape, which can likely be automated via agentic workflows that handle DFT input generation, convergence checking, and landscape construction, making the method easier to extend to new surfaces.^93,94^ Whether the approach reaches the full set of elementary steps, reaction environments, coverages, and electronic or spin reorganization that govern complete catalytic cycles remains open. Nevertheless, feedback from HDRL-FP grounds hypothesis generation in computed energetics, which makes mechanistic alignment of LLMs in catalysis an increasingly practical target.^69,95^

External verification is another route to ground model outputs, but in this case with experimental data and domain knowledge. Reaction steps can be checked for stoichiometry, intermediates for geometric plausibility, and proposed pathways for consistency with energetics, spectroscopy, or reactor-scale observables.^16,69,95^ In more complex systems with dynamic surfaces, coverage-dependent behavior, and multiple active-site ensembles, full verification of the proposed model or parameter may not be possible, but partial verification is still far more informative than none.^16,18,72^ An example that has been explored by our group is phase-selective synthesis of polymorphic catalysts. Transition metal carbides (TMC) are a versatile material class, consisting of iron carbides for Fischer–Tropsch synthesis and water–gas shift,^96^ molybdenum carbide for CO_2_ conversion and methane reforming, and tungsten carbide for hydrogenation, isomerization, and reforming. In all of these cases, the catalysts exhibit polymorphic behavior that is a strong function of synthesis and reaction conditions, resulting in a nontrivial problem for evaluating phase pure carbides.

Tungsten carbide (W_*x*_C) exists in several phases including WC, W_2_C, and nonstoichiometric WC_1–*x*_, and multiple phases may coexist under typical synthesis conditions below the eutectoid temperature of approximately 1250 °C.^47^ Metallic tungsten itself exists as thermodynamically stable α-W (*Im*3̅*m*) and metastable β-W (*Pm*3̅*n*), and the precursor structure influences both carburization kinetics and the resulting carbide phase distribution.^97^

Under typical synthesis conditions below approximately 1000 °C that use CH_4_ as the carbon precursor and WO_3_ as the starting material, the competing phases include thermodynamically stable δ-WC (hexagonal, *P*6̅*m*2), metastable β-W_2_C (orthorhombic, *Pbcn*), and metastable γ-WC_1–*x*_ (cubic, *Fm*3̅*m*). Phase-selective synthesis of δ-WC and β-W_2_C has been demonstrated through control of carburization kinetics and particle size, but achieving high selectivity toward cubic γ-WC_1–*x*_ remains elusive.^47,97^ Phase selectivity depends on precursor identity, metal loading, the support, reduction conditions, heating rate, carburization temperature, synthesis gas composition, and dwell time. Because the synthesis parameter space contains both categorical and continuous variables, exhaustive experimental mapping is intractable, and BO-ICL is a natural fit.

By encoding the materials with natural language, we can incorporate both qualitative and quantitative experimental knowledge in the representation without predefined descriptors. BO-ICL is well suited for this tungsten carbide data set because the design space is high-dimensional and nonlinear, labeled data are sparse, and each evaluation requires significant experimental effort. The LLM contributes chemical reasoning through pretrained knowledge of synthesis-structure relationships, while a physically grounded measurement, here XRD-derived weighted profile *R*-factor (*R*_wp_), a measure of the numerical goodness-of-fit, provides external verification and determines whether each proposed experiment moves the optimization toward the desired phase.

In this phase-controlled tungsten carbide example, the objective function in the BO loop is an unknown function *f*(*x*) over the parameter space Ω, and the optimization problem is

1 $$\arg\;\max_{x\in \Omega }\;f\left(x\right)$$

For phase-selective synthesis, a physically interpretable choice is *f*(*x*) = −*R*_wp_(*x*), where *R*_wp_ is the weighted-profile residual from X-ray diffraction (XRD) data

2 $$R_{wp}={\left[\frac{\sum_{i}w_{i}{\left(y_{i}^{obs}-y_{i}^{calc}\right)}^{2}}{\sum_{i}w_{i}{\left(y_{i}^{obs}\right)}^{2}}\right]}^{1/2}$$

and *y*_*i*_^obs^ and *y*_*i*_^calc^ are the observed and calculated intensities at diffraction point *i*, and *w*_*i*_ are statistical weights. In practice, *R*_wp_ is computed through full-pattern refinement or target-phase reference matching against the desired phase, which in this case can be any desired phase of tungsten carbide. In our workflow, we use GSAS-II crystallographic Rietveld refinement software in the optimization loop so that each experimental diffractogram is automatically converted to a scalar reward signal, *R*_wp_. The utility of our approach is that the objective function of *R*_wp_ quantifies phase selectivity without directly assigning a phase to the unknown experimentally measured structure, but the reward is directly verifiable and is coupled with actionable synthesis methods to facilitate subsequent experiments, as shown in Figure 6. However, in cases where experimentally synthesized phases are amorphous or indistinguishable polymorphs, our approach may not yield sufficient information for successful optimization via BO-ICL. After each evaluation, the observed (*x*, *R*_wp_) pair is added as new context and the objective function, α­(*x*), is recomputed. The workflow then proceeds through the BO-ICL iterative cycle, where the LLM proposes candidate synthesis protocols based on patterns extracted from previously reported experiments, the proposed conditions are evaluated experimentally, and the resulting *R*_wp_ values are returned as new context. Because generalization is driven by the chemical prior encoded during LLM pretraining rather than by task-specific supervised labels, BO-ICL can navigate the high-dimensional W_*x*_C synthesis space without a large labeled data set.

**Figure 6:**
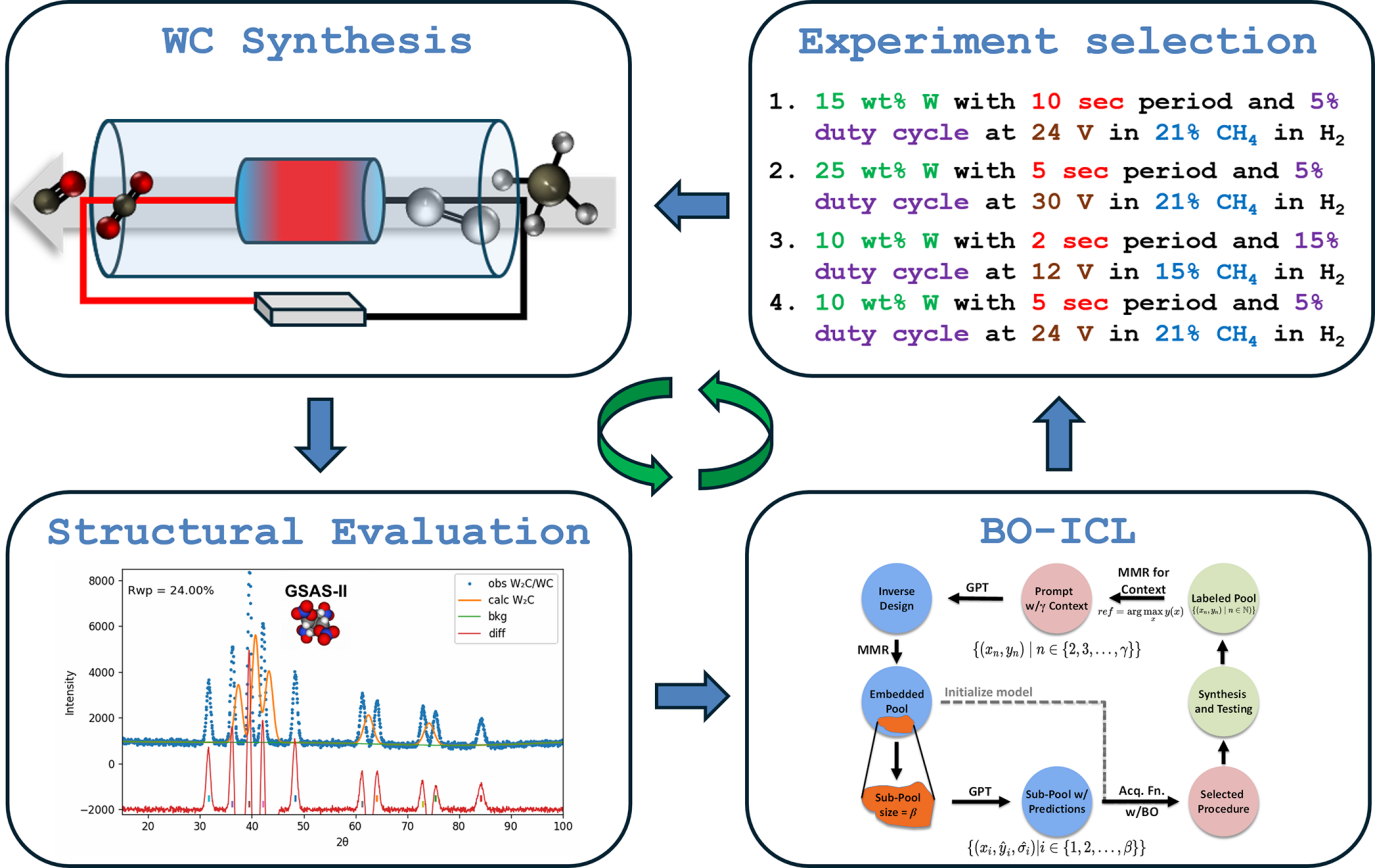
BO-ICL-guided closed-loop optimization of tungsten carbide synthesis. Candidate synthesis conditions are proposed within a Bayesian optimization framework using in-context learning, synthesized, and evaluated by XRD via Rietveld refinement with GSAS-II. The resulting scalar reward signals, *R*_wp_, are then returned to the model to guide subsequent experiment selection. This closed-loop links experiment design, materials synthesis and data-driven structural evaluation for iterative optimization of targeted tungsten carbide phases.

Zeolite synthesis represents another interesting application, although a substantially harder test of the structure optimization framework. Hundreds of distinct framework types are cataloged in the International Zeolite Association (IZA) database, each defined by a unique topology, pore geometry, and T-site connectivity, and many theoretically predicted frameworks remain unsynthesized.^98^ The synthesis parameter space is much higher-dimensionality than tungsten carbides, spanning silica and alumina precursor identity and ratio, structure-directing agent (SDA) chemistry, mineralizer identity and concentration, crystallization temperature and duration, and pH.^99^ The mapping from these parameters to specific frameworks is highly nonlinear, sparsely sampled, sensitive to impurities and environmental variables, and inconsistently reported across studies.^17^ Simulated XRD reference patterns can be generated from hypothetical framework structures, providing the same *R*_wp_-based objective function used in the tungsten carbide example, but the number of competing phases and the sensitivity of selectivity to small parameter changes make the zeolite space more challenging to accurately probe and model.

Building on earlier natural language processing pipelines that extract zeolite synthesis routes directly from the literature,^100^ LLM-based pipelines can now convert dispersed and fragmented synthesis routes into structured representations that encode both quantitative parameters and qualitative constraints, such as tendencies for certain SDAs to result in 10-ring over 12-ring pore systems or for fluoride media to favor pure-silica frameworks.^17,18^ Because the zeolite system is more complex, a two-stage strategy is needed, as demonstrated by an application of the closed-loop autonomous materials exploration and optimization (CAMEO) framework for Ge–Sb–Te materials.^27^ Early iterations of the model start with risk-minimization-based active learning to build a descriptive phase map across the design space, and once the map converges, the system switches to BO-driven optimization near the phase boundaries where selectivity gradients are the steepest. The ZeoSyn data set, which provides synthesis routes across more than 230 framework types, including explicit negative outcomes, enables construction of a synthesis plausibility verifier that applies layered checks on charge balance, SDA geometric compatibility, and kinetic plausibility against literature activation energies.^99,101^ When the verifier rejects a proposed synthesis, that signal can be fed back as a negative reward in an RLVR training loop, connecting domain-specific verification directly to the alignment infrastructure described earlier in this section.^69,102,103^ The fully integrated workflow, combining language-derived priors, active learning, automated XRD refinement, and RLVR-aligned verification, has not yet been demonstrated as a complete system, but the individual components have been validated separately. The zeolite design space possesses the theoretical basis and well-defined characterization targets, making zeolite synthesis a prime example for future testing and optimization.

Coupling language-guided hypothesis generation with tool-mediated verification, physically grounded rewards, and representations that encode physical rather than lexical distance brings both the reasoning process and the metric it operates on into closer conformity with physical reality. Because the two alignment problems are coupled, progress requires advancing them jointly rather than in isolation.

### 3.3 Bridging the Lab-to-Pilot Scale

The preceding sections treat catalyst discovery and phase-selective synthesis as the primary targets for language-guided workflows. An equally important and largely unaddressed challenge in the field is translating lab-scale catalysts to industrially relevant scales, where performance often degrades for reasons that are difficult to anticipate from lab-scale data. Pilot- or industrial-scale testing is unavailable to most researchers, so lab-scale discovery and scale-up are rarely connected in ways that provide fundamental feedback to academia. The following section is a forward-looking analysis by which language-guided workflows could begin to bridge the catalyst development gap between academia and industry.

Two well-documented discrepancies drive the gap between academic and industrial catalysis.^104^ First, technical catalyst formulation requires binders and structural promoters that are not present in lab-scale powder catalysts, and these additives are not necessarily inert. The binders affect stability, crush strength, and pressure drop, but their influence on catalytic performance is rarely studied systematically at the research scale.^104^ Second, the order-of-magnitude difference in reactor diameter between lab-scale (0.25–0.5 in) and industrial-scale (∼6 in) reactors changes the apparent kinetics and heat/mass transfer, and behavior observed in isothermal microreactors does not necessarily transfer to adiabatic packed beds where radial temperature gradients become significant (Figure 7).

**Figure 7:**
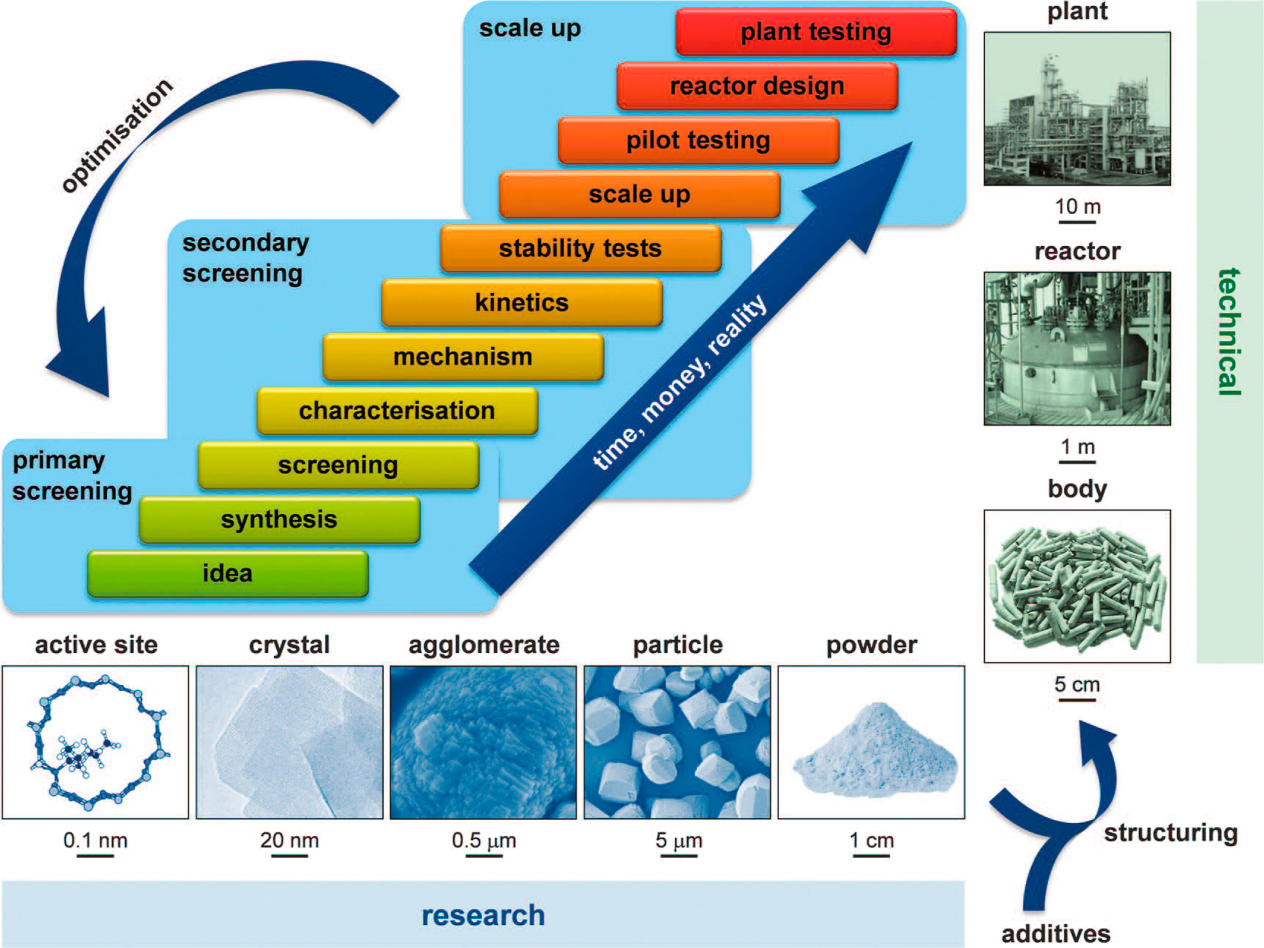
Typical sequence of interactive tasks followed in a catalyst development program. A key step is the translation of a promising research catalyst into a technical catalyst involving the extrapolation of laboratory recipes to an industrial scale and the structuring of powders with additives into mm-sized bodies. Successfully implemented technologies are those which deliver recognizable performance benefits upon bridging the multiple length scales from the active site to the chemical plant. Reproduced with permission from ref 104. Copyright 2013 Royal Society of Chemistry.

Language-based catalyst representations can help bridge this length scale gap because text preserves the process-relevant information that changes across scales. A synthesis procedure written for a gram-scale powder and one written for a kilogram-scale technical catalyst differ in specific and informative ways, including the addition of binders, changes in calcination conditions and heating rates, and differences in pelletization and extrusion steps.^104^ When catalysts are represented as text, these scale-dependent details are part of the representation, rather than discarded during featurization. Reactor configuration, operating conditions, feed composition, and time-on-stream can likewise be encoded directly into the language-based representation, so that reactor topology becomes a learned variable rather than an external assumption.^105,106^

The advantage of language-based representation creates a practical opportunity for closing the loop between lab-scale discovery and pilot-scale validation. The principal bottleneck in catalyst scale-up is the scarcity of linked data across length scales. Lab-scale screening generates thousands of data points under idealized conditions, while pilot-scale testing is generally unavailable to most researchers, meaning that the two data sets are rarely connected in a way that supports systematic learning. An LLM operating over language-based representations can interpret both data sets in a common format, learning correlations between synthesis methods, reactor configurations, and performance metrics that span scales.^107^ When pilot-scale data, including deactivation behavior and postreaction characterization are returned to the model as new context, the model can update its predictions regarding which lab-scale candidates are most likely to seamlessly transition to pilot-scale technical catalysts.

Multiphysics reactor simulations provide an additional verification layer, analogous to how XRD-derived *R*_wp_ provides verification for phase-selective synthesis. Finite-element models that self-consistently couple fluid dynamics, heat and mass transfer, and heterogeneous reaction kinetics can disentangle intrinsic surface activity from transport-induced effects under realistic operating conditions.^95^ The resulting physics-informed digital twin assimilates pilot-scale data to recalibrate kinetic and transport parameters and reduces uncertainty in subsequent iterations. When the LLM proposes a candidate catalyst and a set of operating conditions, the reactor model can evaluate whether the predicted performance is physically achievable at the target scale before researchers commit to an expensive pilot-scale experiment. This stepwise verification, first checking intrinsic catalytic properties against thermodynamic and kinetic criteria, and then scale-dependent behavior against reactor-level transport models, is the scale-up analog of RLVR described above.

Industrial reactor feeds introduce an additional source of complexity that language-based representations are well suited to address. Academic studies typically use pure feeds, but industrial CO_2_-derived streams contain contaminants such as H_2_S, SO_*x*_, and NO_*x*_ that can poison active sites, promote overhydrogenation, or accelerate deactivation. These effects are feed-specific and difficult to predict from first principles.^65^ By encoding feedstock composition directly in the text-based representation, the LLM can learn correlations between contaminants and catalytic deactivation across catalysts and reaction conditions. In some cases, contaminants that are expected to be harmful have unexpected benefits. For example, H_2_S improves alcohol synthesis performance over Co–Mo–S catalysts from biomass-derived syngas.^108^ A language-based model trained on sufficiently diverse feed compositions will capture these nonobvious interactions without requiring a mechanistic model of each poisoning pathway.

Catalyst deactivation poses a further barrier to bridging the lab-to-pilot gap, and it is poorly captured by the short experiments that dominate AI-enabled high-throughput discovery. Commercially relevant catalyst lifetimes are measured in years, and the dominant deactivation pathways for industrial catalysts, including copper sintering and poisoning in methanol synthesis, evolve slowly and nonlinearly.^109,110^ Establishing commercial relevance therefore requires hundreds to thousands of hours on stream, both to reach representative end-of-life states and to characterize the deactivation mechanisms. Recent work tracing CuZnO deactivation from the micro to the macro scale clearly illustrates that plant-scale behavior depends on mechanistic details that are not apparent from short experiments.^111^

Because full-lifetime testing is impractical, industrial practice relies on accelerated-aging protocols that are calibrated to reproduce the mechanisms observed under real conditions and validated against pilot-scale reactors.^110^ This industrial reality imposes a specific limit on language-guided, high-throughput workflows, because in this case, screening throughput is bounded by the rate-determining step of long-duration time-on-stream testing. Therefore, stability testing rather than synthesis or initial activity becomes the bottleneck, and the throughput advantage of these AI-enabled workflows is lost.^112^ The value of LLMs for catalyst deployment therefore depends on whether deactivation can be extrapolated from short and accelerated tests, a problem well suited to a language-based representation that encodes accelerated-aging conditions, postreaction characterization, and time-on-stream trajectories in a common format and relates them to end-of-life performance.^111^

The scale-up challenge is ultimately a data-integration problem. Lab-scale discovery, technical catalyst formulation, pilot-scale testing, and reactor modeling each generate data in different formats, at different volumes, and with different levels of uncertainty. Language-based representations provide a common encoding that can accommodate all of these data without requiring a unified set of descriptors. The value of this integration depends on whether the resulting predictions are validated at each stage, through reproducibility across laboratories, consistency with reactor simulations, and long-duration pilot testing under representative conditions. When the appropriate validation infrastructure is in place, language-guided workflows can compress what has traditionally been a multiyear scale-up campaign into a substantially shorter and more efficient process, while generating transferable knowledge about which synthesis-structure–performance relationships hold across length scales.

## 4 Conclusions

LLMs are reshaping how researchers access and interpret catalysis data by lowering the barrier to hypothesis generation, organizing dispersed knowledge from the literature, and encoding chemical intuition in a form that can be used in closed-loop workflows. The most reliable demonstrations to date couple language-based reasoning with physics-grounded feedback, rather than treating model outputs as end points. The discussions within this perspective, including data integration, representation methods, and the absence of standardized benchmarks, are structural rather than incidental. Heterogeneous catalysis lacks the conditions under which AI has advanced most rapidly, namely abundant labeled data, compact representations, and well-defined ground truths. The field of catalysis therefore places implicit rules on how language models are deployed, and reinforcement learning from verifiable rewards, domain-specific rubrics, and tool-integrated workflows are the infrastructure needed to reward correctness over fluency. The opportunity of LLMs for catalysis is not simply accelerated catalyst screening, but the construction of workflows to generate hypotheses, validate experiments, and structure data in a format that is accessible and verifiable. The field is primed for a rapid step-change where language-based approaches become a significant part of generation and verification of fundamental catalytic knowledge.
